# MAPK Signaling Pathway May Directly Regulate the Expression of Hydrophobin Genes in *Flammulina filiformis*

**DOI:** 10.3390/jof12040268

**Published:** 2026-04-08

**Authors:** Qianhui Huang, Zongjun Tong, Xiaoling Guan, Qiongxuan Qiao, Shengrong Liu, Weirui Zhang, Qi Wei, Baogui Xie

**Affiliations:** 1 College of Biosience and Engineering, Ningde Normal University, Ningde 352100, China; elberthuang123@163.com (Q.H.); 19905052503@163.com (X.G.); m15835303932@163.com (Q.Q.); fjhost@163.com (S.L.); 2 Fujian Higher Education Research Center for Local Biological Resources, Ningde 352100, China; 3 Institute of Edible Fungi, Shanghai Academy of Agriculture Sciences, Shanghai 201304, China; ttzjun1@163.com; 4 Mycological Research Center, Fujian Agriculture and Forestry University, Fuzhou 350002, China; mrcfafu@163.com

**Keywords:** aerial hyphae, hydrophobin, MAPK signaling pathway, filamentous growth, *cis*-regulatory element

## Abstract

Fungal hydrophobins reduce the surface tension of hyphae so that hyphae can grow into the air. Reduced expression of hydrophobin genes results in abnormal morphogenesis of both hyphae and the fruiting body of *Flammulina filiformis*. Previous studies showed that filamentous-growth MAPK signaling pathway directly modulates pseudohyphae formation in budding yeast, so we hypothesized that the specific transcription factor in this pathway may also directly regulate the expression of hydrophobin genes in *F. filiformis*. Downstream of the G protein, the cAMP/PKA signaling pathway is parallel with the filamentous-growth MAPK signaling pathway in regulating the filamentous growth of fungi. Thus, the cAMP addition test was carried out to exclude the involvement of the PKA/cAMP signaling pathway in aerial-hyphae deficiency of the three mutants used in our previous study. Transcriptomic analysis showed common changes in the MAPK signaling pathway of the three mutants, including 6 downregulated and 3 upregulated genes in common. Transcription factor Tec1 was one of the upregulated genes, and it is a pathway-specific transcription factor for filamentous growth. Motif prediction showed that putative binding sites of Tec1 and Ste12 existed in the promoter region of the three chosen hydrophobin genes mentioned in our previous study, and DAP-seq analysis suggested that putative binding sites of Tec1 and Ste12 were located in 10 hydrophobin genes, respectively, and there were 8 in common for both the transcription factors. These results gave suggestive evidence supporting our hypothesis. We have identified a potential regulatory connection between the filamentous-growth MAPK signaling pathway and hydrophobin genes through Tec1 and Ste12. However, functional validation is required to confirm direct regulation between both the transcription factors and the downstream genes.

## 1. Introduction

Hydrophobins are secreted structural proteins that can self-assemble on the surface of the cell wall to reduce the surface tension of hyphae, so that hyphae can grow into the air [1]. Hydrophobins exist in filamentous fungi from basidiomycetes, ascomycetes, and zygomycetes, and hold multiple important functions during the life cycle of fungi [2,3]. There are two types of hydrophobins, known as class I and II hydrophobins. Class I hydrophobins exist in both basidiomycetes and ascomycetes, while class II hydrophobins only exist in ascomycetes [4], and they have been evolving separately [5]. Hydrophobins participate in many important biological processes in fungi, such as morphogenesis [6], invasion [7], sexual development [8], and stress tolerance [9]. *Aspergillus fumigatus RodA* knockout mutant showed decreased invading ability, while RodA in *Aspergillus nidulans* affects sexual development [10,11]. Hydrophobins showed even more significant influence on the morphogenesis of mushrooms. *Schizophyllum commune Sc3* knockout mutant could not produce aerial hyphae, with its hyphae tightly stuck to the medium surface [12]. In *S. commune*, Sc7 and Sc14 are two fruiting body-specific hydrophobins, which would not be expressed in hyphae before the fruiting body develops [13]. Hyd9, a hydrophobin from *Flammulina filiformis*, bears a more decisive role than other hydrophobins in the morphogenesis of this mushroom, for its RNAi (RNA interference) mutants showed impressive flaws in the development of both aerial hyphae and fruiting body [14]. Hyd9 and Sc3 are both Class I hydrophobins, and severe reduction of either gene’s expression results in loss of aerial hyphae. However, the regulatory mechanisms underlying their expression differ, i.e., Sc3 is expressed specifically in aerial hyphae, whereas Hyd9 is highly expressed in both hyphae and fruiting bodies [12,15]. So, uncovering the regulation mechanisms of hydrophobin expression in *F. filiformis* holds profound meaning in understanding filamentous growth and mushroom production.

Several factors regulate the expression of hydrophobin genes, such as mating, nutritional conditions, and light [16,17,18]. However, a clear pathway through which the expression of hydrophobin genes was regulated is still unknown. Previously, a *thn* gene mutant of *S. commune* showed severe reduction of aerial hyphae. These phenotypes were the same as the phenotypes of *F. filiformis* mutants in our previous study [5]. Scholars finally found that the *thn* gene could regulate the expression of the *Sc3* gene, which encodes a hydrophobin [12,19]. The *thn* gene encodes a regulator of G-protein signaling (RGS), a vital component in G-protein signaling. In ascomycetes, RGS regulator knockout mutants also showed significantly inhibited expression of hydrophobin genes [20,21]. Downstream the RGS, protein kinases in the MAPK (mitogen-activated protein kinase) signaling pathway can also regulate the expression of hyhrophobin genes, such as LaeA, LDB1, Hog1, tvk1 [22,23,24,25]. Furthermore, transcription factor FL in the MAPK signaling pathway played an important role in regulating the expression of hydrophobin genes, and FL was demonstrated to bind to hydrophobin gene *eas* by the *cis*-acting element 5′-CGG(N)9CCG-3′ [26]. However, there is no homologue of FL in basidiomycetes. Scholars tried to explore the transcription factor directly regulating hyhrophobin genes through the upstream *cis*-acting elements, and found a BRE (brlA response element) element in the promoter region of *Sc3* gene, which is the *cis*-acting element of *A. nidulans* transcription factor brlA [27]. However, they ignored whether this BRE element existed in the promoter region of other hydrophobin genes or not. Moreover, the system regulating growth and development in basidiomycetes is obviously different from that in filamentous ascomycetes, for example, homologues of *Aspergilus* regulators abaA, brlA, flbC, flbD, fluG, phiA, stuA, and wetA are absent in basidiomycetes [28]. How signaling passes to hydrophobin genes downstream of Thn in basidiomycetes is still unclear. Downstream of the G protein, the cAMP/PKA signaling pathway (cyclic AMP and the cAMP-dependent protein kinase signaling pathway) is parallel with the filamentous-growth MAPK signaling pathway in regulating the filamentous growth of fungi [29]. If the components in this pathway were mutated, such as adenylyl cyclase or Gpa2, a G protein subunit, extracellular cAMP could restore fungal morphogenesis by normalizing development-related gene expression [30,31].

In our previous study, we demonstrated that the aerial-hyphae-deficient morphology was caused by the extremely inhibited expression of hydrophobin genes in *F. filiformis* [5]. Here, in order to reveal the regulatory relationship between hydrophobin genes and its upstream signaling pathway, firstly, cAMP addition test was carried out to check whether components in cAMP signaling pathway change or not; secondly, comparative transcriptome analysis was performed to show gene expression in MAPK signaling pathway in the mutants; finally, putative *cis*-acting elements of hydrophobin genes were predicted, and DAP-seq (DNA affinity purification sequencing) was subsequently used to demonstrate the regulatory relationship between Tec1, Ste12 and hydrophobin genes.

## 2. Materials and Methods

### 2.1. Fungal Strains

Three aerial-hyphae-deficient mutants, UV-70, UV-104, and UV-128, were obtained from UV-radiated oidia spores of WT583, a monokaryotic *F. filiformis* strain. These mutants were stored in the Mycological Research Center of Fujian Agriculture and Forestry University.

### 2.2. cAMP Addition Test

cAMP addition can restore the mutants’ morphology and promote the filamentous growth if the genes in the cAMP/PKA signaling pathway were mutated. According to previous research [31], the final density of cAMP in the medium was 1 mmol/L in the treatment group, and no cAMP was added in the control group.

### 2.3. Sample Collection and RNA Sequencing

Because the mutants produced rare aerial hyphae, cellophane was used to collect hyphae samples. Spawn was inoculated on the sterile cellophane so that hyphae could grow separately from the medium in the plates. This operation provided convenience for collecting hyphae samples. The mutants grew so slowly and nonuniformly that sample collection was performed almost one month after inoculation. Three biological replicates were collected for each mutant. The collected samples were sent to Novogene Bioinformatics Institute (Beijing, China) for RNA sequencing. RNA extraction and quality determination, construction of transcriptome sequencing library, sequencing, and quality control of reads were performed according to the standard procedure of Novogene Bioinformatics Institute [32]. After clean reads were obtained, the FPKM (fragments per kilobase of exon model per million mapped fragments) value of the transcripts was calculated according to a previously published method [33].

### 2.4. KEGG Annotation of Differentially Expressed Genes (DEGs)

DEGs were chosen according to the filtering standard described by Chen et al. [34], i.e., FPKM > 1, |log_2_FC| > 2, *p* < 0.05, and *FDR* < 0.05. Corresponding protein ko numbers of DEGs were gained by Batch Functional Annotation of eggNOG-mapper v2 (http://eggnog6.embl.de/#/app/home, accessed on 12 November 2025), then, the OMICSHARE tool (https://www.omicshare.com/tools/, accessed on 13 November 2025) was applied to visualize KEGG pathway distribution of DEGs.

### 2.5. RT-qPCR and Data Analysis

Sample collection was the same as mentioned above. Total RNA extraction and PCR reaction were carried out based on the protocol employed by Huang et al. [35]. PrimerQuest Tool (https://sg.idtdna.com/PrimerQuest/Home/Index, accessed on 20 May 2020) was used for designing primers of DEGs. Primer length was set from 20 nt to 24 nt, and Tm between 60 and 63 °C. The amplified length spanned from 70 bp to 150 bp, and other parameters were set as default. The glyceraldehyde-3-phosphate dehydrogenase gene of *F. filiformis* was used as the internal control. All primers are listed in Supplementary Material S1. The 2^−ΔΔCT^ method was employed to analyze the RT-qPCR data [36].

### 2.6. Predicting Potential Transcription Factor of Hydrophobin Genes

According to the gtf (gene transfer format) file of genome annotation, the 1000 bp promoter sequences upstreaming the start codon of the chosen hyhrophobin genes were taken from the *F. filiformis* genome, then, the common conserved motifs in the 1000 bp promoter regions were visualized by MEME (http://meme-suite.org/tools/meme, accessed on 24 August 2025). Finally, these motifs were compared with the *cis*-acting elements of Ste12, Tec1, and SKO1, et al., which are transcription factors in the yeast MAPK signaling pathway. The transcription factor provided high similarities between its cis-acting elements and the predicted motifs was considered as the possible one.

### 2.7. DAP-Seq of F. filiformis Tec1 and Data Analysis

DAP-seq was performed by IGENEBOOK Biotechnology (Wuhan, China) according to the previously published protocol [37]. Firstly, *F. filiformis* genomic DNA library was built using a NEBNext^®^ DNA Library Prep Master Mix Set for Illumina (New England Biolabs, lnc., Ipswich, MA, USA). Secondly, the Halo Tag expression plasmid of *fftec1* was constructed by fusing the CDS of *fftec1* into the pFN19K HaloTag T7 SP6 Flexi Vector (Promega, Madison, WI, USA), then the recombinant plasmid was transferred into TnT SP6 High-Yield Wheat Germ Protein Expression System (L3260, Promega) for fusion protein production. The Magne HaloTag Beads were used to purify the fusion protein, then the beads and protein mixture were directly incubated with 500 ng genomic DNA library with slow rotation for 1 h at room temperature. Finally, non-specific bound DNA fragments were removed by washing several times, and the purified DNA fragments were sequenced on Illumina NovaSeq 6000 with the PE 150 method. In order to eliminate the background influence of fungal genome on peak calling, the same genomic DNA library was prepared as the input control. To obtain more precise data, DAP-seq analysis of *Ste12* was also performed. DAP-seq of both transcription factors was repeated twice.

For data analysis, Trimmomatic (version 0.36) was used to eliminate low-quality reads [38]. Clean reads were mapped to the *F. filiformis* genome by the BWA software (version 0.7.15) [39]. MACS2 software (version 2.1.1.20160309) was employed to call peaks by default parameters (bandwidth, 300 bp; model fold, 5, 50; FC, 2; q value, 0.001) [40]. HOMER (version 3) was used to predict motif occurrence within peaks with default settings for a maximum motif length of 12 base pairs [41]. ClusterProfiler (version 4.2.2) in the R package was employed to perform KEGG (Kyoto Encyclopedia of Genes and Genomes, http://www.genome.jp/kegg/) enrichment analysis, and the KEGG enrichment analysis was calculated using a hypergeometric distribution with a q-value cutoff of 0.05 [42].

## 3. Results

### 3.1. cAMP Addition Failed to Fix Aerial-Hyphae-Deficient Mutants

**Figure 1:**
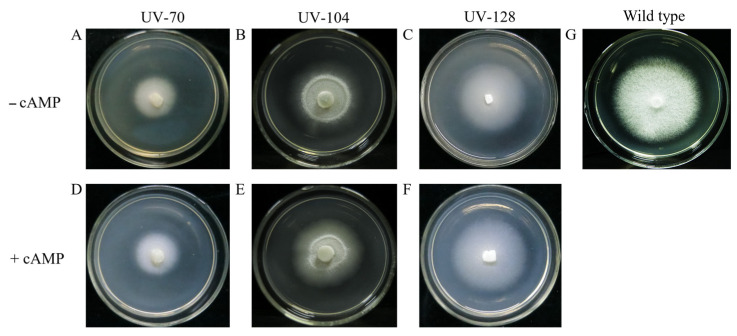
cAMP addition test of the mutants. The three aerial-hyphae-deficient mutants are UV-70, UV-104, and UV-128, previously used in our study [5]. No cAMP was added in control group (**A**–**C**), 1 mmol/L cAMP was added in treatment group (**D**–**F**). The monokaryotic strain WT583 ((**G**), wild type) was used as positive control.

Based on our previous study on the aerial-hyphae-defecient mutants [5], a cAMP addition test was carried out to check whether the phenotype of the mutants would be restored or not. Obviously, hyphae of the three mutants in the plates with cAMP seemed the same as those in the plates without cAMP (Figure 1A–F). So, it was reasonable to propose that the phenotype of the mutants could not be resumed by exogenous cAMP, and the aerial-hyphae-deficient phenotype may not be caused by the cAMP/PKA signaling pathway.

### 3.2. KEGG Pathway Annotation of DEGs

**Figure 2:**
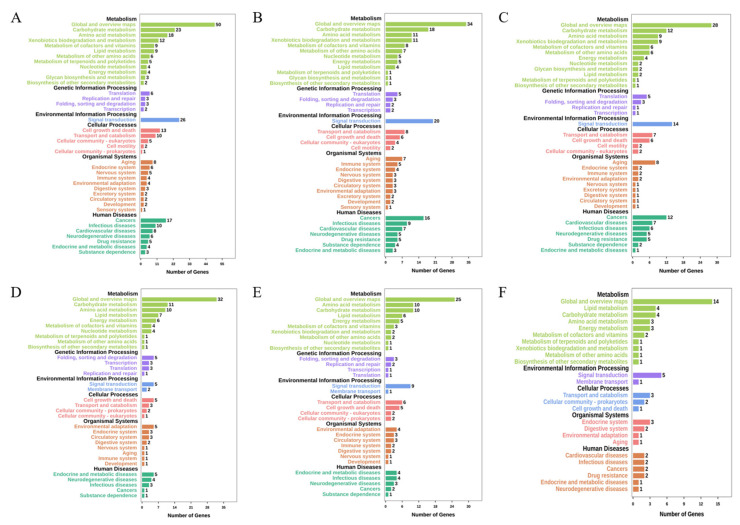
KEGG pathway analysis of DEGs. KEGG pathway analysis of downregulated DEGs of UV-70, UV-104, UV-128 (**A**–**C**), and upregulated DEGs (**D**–**F**), respectively.

1745, 973, 802 downregulated and 609, 540, 329 upregulated genes were detected in UV-70, UV-104, and UV-128, respectively. Only 107, 84, 62 downregulated and 56, 48, 22 upregulated genes were annotated in KEGG (Supplementary Material S2). There were 26, 20, 14 downregulated genes in the item signal transduction of UV-70, UV-104, UV-128, respectively, among which, 12 (46%), 12 (60%), 6 (43%) genes were distributed in the MAPK signaling pathway (Figure 2A–C). 5, 9, 5 upregulated genes were annotated in the item signal transduction of UV-70, UV-104, UV-128, respectively, of which 3 (80%), 6 (67%), 3 (80%) genes belonged to the MAPK signaling pathway (Figure 2D–F). These data indicated that, in UV-70, UV-104, and UV-128, both downregulated and upregulated genes in the MAPK signaling pathway held the highest rates among all signaling pathways in the item signal transduction.

DEGs distributed in the MAPK signaling pathway were listed in Table 1 and Table 2. The downregulated genes outnumbered the upregulated genes in the MAPK signaling pathway of the three mutants. Among the downregulated genes, there were 6 common genes, of which 5 belonged to the MAPK signaling pathway—yeast (ko04011). MSTRG5365.1, MSTRG10940.1, and MSTRG11179.1 were annotated as STE3, a pheromone receptor of fungi. STE3 locates on the cell membrane and transfers signals inside by catalyzing GDP to GTP. MSTRG7679.1 was annotated as ANP1, a MAPKK kinase mainly responding to stress in the plant MAPK signaling pathway [43]. ANP1 has no homologue in yeast; however, its location in the plant MAPK signaling pathway was the same as that of STE11 in yeast, and its overexpression could fix the MAPK signaling in the yeast STE11 mutant [44]. MSTRG10971.1 was annotated as CK1 (casein kinase 1). Its yeast homologue Yck1 regulates several important processes, including nutrient sensing, cell growth, budding, and cell division [45,46,47]. MSTRG10559.1 was annotated as SKO1, a transcription factor mainly involved in stress response [48,49]. Of the upregulated genes, there were 3 common genes, of which 2 belonged to the MAPK signaling pathway—yeast (ko04011). MSTRG 472.1 was annotated as a transcription factor belonging to the TEAD family, and its homolog in yeast is Tec1, which directly regulates the expression of genes related to filamentous growth [50]. MSTRG.2205.1 was annotated as BCK1, also a MAPKK kinase, and its location in the MAPK signaling pathway is the same as that of STE11 and ANP1. The difference is that BCK1 is located in the MAPK signaling pathway responsible for cell wall integrity [51,52]. MSTRG.4611.1 was annotated as a serine/threonine-protein phosphatase 5 (PPP5C), the function of which was rarely explored in filamentous fungi; however, research showed PPP5C was involved in glucose homeostasis in mouse cells [53].

### 3.3. Expression of Common DEGs in MAPK Signaling Pathway

**Figure 3:**
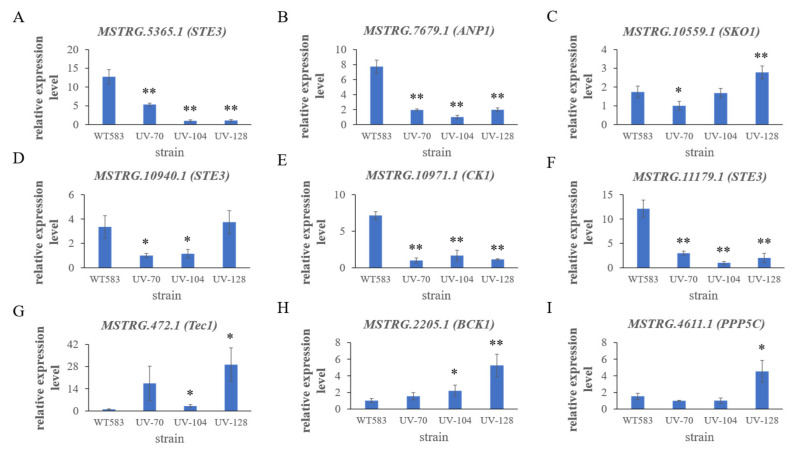
Relative expression levels of the common DEGs in MAPK signaling pathway. (**A**–**F**) are downregulated DEGs, and (**G**–**I**) upregulated DEGs. The title of each subfigure is represented by the DEG names in this study and their corresponding homologues from budding yeast (in the parentheses). Statistical significance was determined by Least Significant Difference method in One-Way Analysis of Variance (* *p* < 0.05, ** *p* < 0.01).

The relative expression levels of the 9 mutual DEGs in the mutants were verified by RT-qPCR (Figure 3). Among the downregulated DEGs, the expression levels of *MSTRG.5365.1*, *MSTRG.7679.1*, *MSTRG.10971.1*, and *MSTRG.11179.1* were all downregulated in all mutants (Figure 3A,B,E,F), while the expression level of *MSTRG.10559.1* was only downregulated in mutant UV-70 (Figure 3C), and there was no significant difference in the expression levels of *MSTRG.10940.1* between WT583 and UV-128 (Figure 3D). Of the upregulated transcripts, the expression levels of *MSTRG.472.1* and *MSTRG.2205.1* were significantly upregulated in mutants UV-104 and UV-128 (Figure 3G,H) and the expression level of *MSTRG.4611.1* was only upregulated in UV-128 compared with WT583 (Figure 3I).

### 3.4. Possible Transcription Factors of Hydrophobin Genes

**Figure 4:**
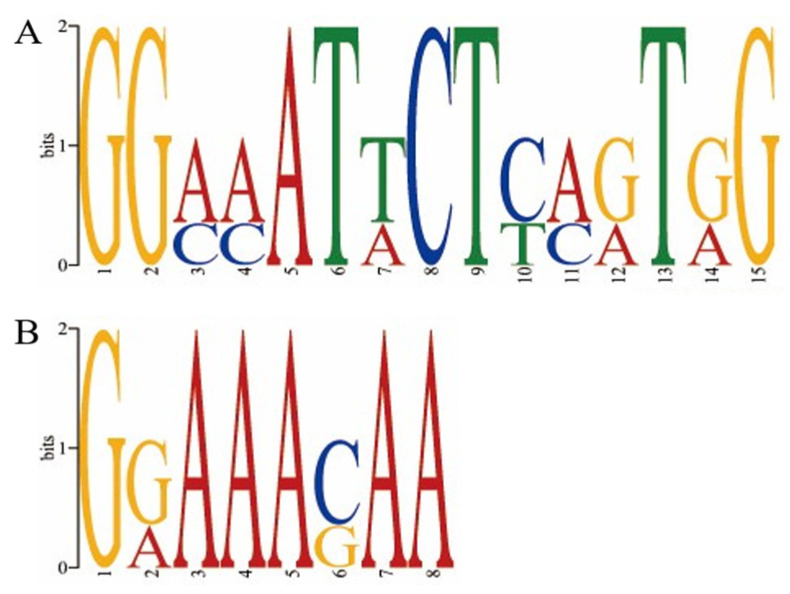
Conserved motif prediction of the upstream sequences of the hydrophobins. Motif (**A**) contains the consensus sequence ATTC recognized by yeast Tec1, and motif (**B**) shows high similarities with the *cis*-acting element of yeast Ste12.

Since Tec1, together with Ste12, can directly regulate the expression of genes related to filamentous growth (https://www.kegg.jp/pathway/sce04011, accessed on 28 November 2025), we hypothesized that it can also directly regulate the expression of hydrophobin genes in *F. filiformis*. The three most downregulated hydrophobin genes in our previous study [5], *MSTRG.6832.1*, *MSTRG.7580.1,* and *MSTRG.8862.1* were chosen for conserved motif detection. Two putative conserved motifs were found holding high similarity with the *cis*-acting elements of Tec1 and Ste12 (Figure 4A,B), which are two important transcription factors regulating filamentous growth of fungi. However, there were no putative conserved motifs of SKO1 (MSTRG.10559.1). There were 1 to 2 mismatches between the putative conserved motifs and the *cis*-acting elements of Tec1 and Ste12 from budding yeast (Table 3).

### 3.5. Determination of the Regulatory Relationship Between Tec1 and Hydrophobin Genes by DAP-Seq

**Figure 5:**
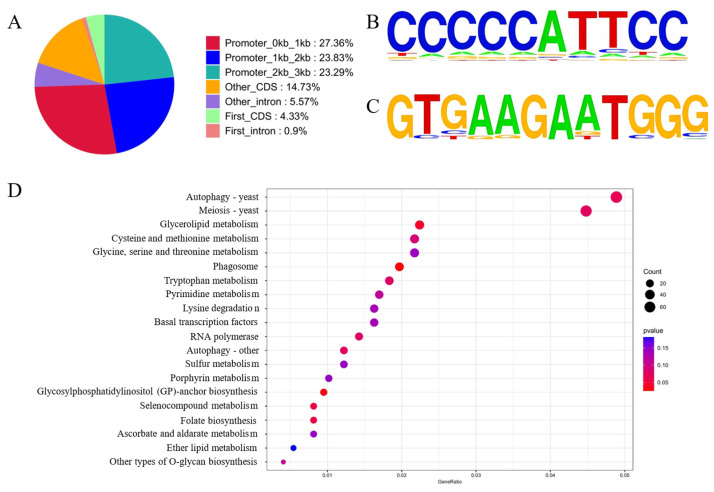
DAP-seq analysis of Tec1 transcription factor. Most peaks located in promoter region, and other peaks in CDS and intron regions (**A**). HOMER software (version 3) predicted conserved motifs and aligned motifs to the motif database. One putative motif was obtained for Tec1 and Ste12, respectively (**B**,**C**). KEGG enrichment of DEGs whose promoter regions were targeted (**D**).

74.48% of the Tec1 peaks were located in the promoter region (0 to 3 kb relative to the transcription start site), while peaks in the first CDS, other CDS, the first intron, and other intron held a much smaller proportion (Figure 5A). Due to transcription factors binding to the promoter region, genes with peaks located in the promoter region were considered as putative targets. Among these promoter peaks, two putative *cis*-acting elements of Tec1 were found (Figure 5B,C). Sequence CCCCCATTCC (Figure 5B) contained a consensus motif ATTC specific for TEAD1 from eukaryotes (e-value 1 × 10^−20^), while sequence GTGAAGAATGGG (Figure 5C) was also predicted to contain a motif for yeast Tec1 (e-value 1 × 10^−10^). KEGG analysis of genes, of which the peaks lay in promoter regions, was performed (Figure 5D). The results showed that most of the top 20 items were metabolism-related. However, it was notable that there were 3 items related to autophagy and 1 item related to meiosis. As to the hydrophobin genes we are concerned, both Tec1 and Ste12 showed binding peaks in the promoter region of 10 hydrophobins, respectively, of which 8 genes were mutual (Table 4). Moreover, both transcription factors showed binding peaks in promoter regions of many other gene families (Supplementary Material S3).

## 4. Discussion

Downstream of the G protein, the MAPK signaling pathway and the cAMP/PKA signaling pathway can both transfer signals to regulate the filamentous growth of fungi [30]. cAMP addition test demonstrated that the cAMP/PKA signaling pathway may not cause aerial-hyphae deficiency in the three mutants. So, we proposed that the factors causing aerial-hyphae deficiency may exist in the MAPK signaling pathway. The subsequent transcriptome analysis focused on the members in the MAPK signaling pathway, and the results showed that interesting expression shifts happened in this signaling pathway of the three mutants. Also, we speculated that this pathway may mainly regulate the expression of hydrophobin genes, which are a decisive factor for aerial hyphae formation. However, which transcription factor in the MAPK signaling pathway can directly activate the transcription of these hydrophobin genes? Answering this question means building a bridge between the MAPK signaling pathway and the downstream regulated genes.

There are several transcription factors in the MAPK signaling pathway. For example, MCM1 is a transcription factor specific for pheromone-related signal transduction, and SKO1 (MSTRG.10559.1) is mainly involved in stress response. However, we are very interested in Tec1. Firstly, Tec1 mainly participates in mycelial formation, pathogenesis, and interaction with other regulatory elements in ascomycota, but its function in basidiomycota is rarely revealed [56]. Secondly, Tec1 functions in parallel with MCM1 but specifically responds to starvation. MCM1, together with Ste12, can activate the expression of genes involved in fruiting body and basidiospore formation, while Tec1 binding to Ste12 can regulate gene expression involved in filamentous growth in ascomycota [30,57]; thus, the MAPK signaling pathway Tec1 lies in is also named filamentous-growth MAPK signaling pathway [30,58]. Our DAP-seq analysis indicated that Tec1 and Ste12 had many targeted genes or gene families in common, including hydrophobin genes. Thirdly, the expression of a structural protein, FLO11, which promotes the formation of pseudohyphae development, is directly activated by Tec1 in *S. cerevisiae* [58,59]. FLO11 and hydrophobin are structural proteins needed for hyphal growth. Based on the facts above, it is rational to hypothesize that Tec1 in *F. filiformis* may directly regulate the expression of genes specific for aerial-hyphae morphogenesis, including hydrophobin genes. Many motifs were predicted in the promoter region of the chosen hydrophobin genes. More interestingly, one putative *cis*-acting element for Ste12 and Tec1, respectively, was included in the predicted motifs, which coincides with the fact that Tec1, together with Ste12, can activate the expression of hyphae-specific genes [50,55,60]. We noticed that there were 1 to 2 mismatches between some predicted motifs and the consensus sequences of cis-acting elements from ascomycota, which we thought may not influence the recognition and binding by transcription factors according to previous research [50,54]. Even in different strains of *Lactobacillus plantarum*, three or more mismatches exist in the consensus sequences of binding sites of the same transcription factor [61].

The transcription factor SKO1 from the MAPK signaling pathway(osmolarity stress responding) also grabbed our attention, for its expression tendency was opposite to that of Tec1 in our study. We noticed SKO1 is a versatile transcription factor involved not only in osmolarity stress, but also in oxidative stress response, and it acts as a transcription repressor for hyphae-specific genes [62,63]. So, its function goes antagonistically with that of Tec1 in filamentous growth, so it is reasonable that the downregulation of SKO1 and the upregulation of Tec1 may not be a coincidence. The mutants failed to form normal aerial hyphae to obtain enough oxygen, so the hypoxia stress induced the expression shifts of both the transcription factors to produce more aerial hyphae. This speculation could explain that there were a few aerial hyphae in the center of the mutant colonies after long-term culture.

But how could Tec1 be upregulated in this study? The previous study showed that Tec1 could be self-activated by binding to its promoter region containing the AGATTCTT sequence element in budding yeast [50]. We then checked the promoter region 1000 bp relative to the translation start site of *Tec1*, and found two putative binding sites for Tec1 itself (see Supplementary Material S4). So it was possible that Tec1 would be activated by itself in *F. filiformis* under hypoxic stress.

In addition to the above transcription factors, the 4 downregulated pheromone receptors appealed to us. MSTRG.5365.1, MSTRG.7714.1, MSTRG.10940.1, and MSTRG.11179.1 are pheromone receptor Ste3.s4, Ste3.s1, Ste3.s3, and Ste3.s5, respectively. Ste3.s3 and Ste3.s5 are non-mating type pheromone receptors. Such pheromone receptors cannot be activated by pheromone and cannot activate sex development [64]. According to an earlier study, many G-protein-coupled receptors do not need specific ligands for signal transduction [65]. Literature showed non-mating type receptor may function by self-recognition [66]. In our study, wild type WT583 is a monokaryon strain, and two types of pheromone receptors are expressed. So non-mating type receptors may work in a pheromone-independent manner without the activation of pheromone. The identification and characterization of the 4 pheromone receptors were seen in the Supplementary Material, identification and characterization of STE3.

A large fraction of putative Tec1 binding sites were located in the promoter region, and putative binding sites in 0 to 1 kb relative to the transcription start site were higher than those in 1 to 2 kb and 2 to 3 kb, respectively. Distribution of *cis*-acting elements in ascomycete fungi showed regular patterns, i.e., mainly in 0 to 1 kb regions [67]. However, no such study was performed in higher fungi, including mushrooms. So we would not have a further discussion on this result. The predicted Tec1 binding motifs were not totally the same with the *cis*-acting elements of Tec1 from yeast and higher eukaryotes [50,54], but they contained the consensus sequence ATTC. Fluctuation in the flanking sequences or consensus sequences is compatible with transcription factor binding [61]. So the differences between our prediction and *cis*-acting elements from other species seem normal.

## 5. Conclusions

This study investigated the regulatory connection between the filamentous-growth MAPK signaling pathway and hydrophobin genes in *F. filiformis*. Our key findings include the following: cAMP addition failed to restore the mutants’ phenotypes; transcriptomic analysis and RT-qPCR revealed common alterations in the filamentous-growth MAPK signaling pathway; motif prediction and DAP-seq analysis identified putative binding sites of Tec1 and Ste12 in the promoter region of hydrophobin genes and many other gene families. These results suggest a potential regulatory relationship between the filamentous-growth MAPK signaling pathway and hydrophobin genes through Tec1 and Ste12. Moreover, this study may possibly represent a mutual regulatory mechanism for hyphal development in all basidiomycetes. However, functional validation through overexpression and RNAi is needed to confirm direct transcriptional regulation and establish a causal relationship between the filamentous-growth MAPK signaling pathway and its downstream genes. Understanding this regulatory network has prominent implications for fundamental fungal development biology and mushroom production.

## Figures and Tables

**Table 1:** Function annotation of the downregulated DEGs in MAPK signaling pathway.

Strains	Transcript ID	Pathway ID	Function Description
UV-70	MSTRG.10971.1	ko04011	CSNK1, CKI; casein kinase 1
	MSTRG.9623.1	ko04011	CDC28, CDC2; cyclin-dependent kinase
	MSTRG.7714.1	ko04011	STE3; pheromone receptor
	MSTRG.5365.1	ko04011	STE3; pheromone receptor
	MSTRG.11179.1	ko04011	STE3; pheromone receptor
	MSTRG.10940.1	ko04011	STE3; pheromone receptor
	MSTRG.3411.1	ko04011	YWHAE; 14-3-3 protein epsilon
	MSTRG.10559.1	ko04011	SKO1, ATF1, PCR1; ATF/CREB family transcription factor
	MSTRG.2606.1	ko04011	STE11; mitogen-activated protein kinase kinase kinase
	MSTRG.3829.1	ko04011	GRE2; NADPH-dependent methylglyoxal reductase
	g5646.t1	ko04010	PKA; protein kinase A
	MSTRG.7679.1	ko04016	ANP1; mitogen-activated protein kinase kinase kinase ANP1
UV-104	MSTRG.10971.1	ko04011	CSNK1, CKI; casein kinase 1
	MSTRG.9623.1	ko04011	CDC28, CDC2; cyclin-dependent kinase
	MSTRG.10940.1	ko04011	STE3; pheromone receptor
	MSTRG.11179.1	ko04011	STE3; pheromone receptor
	MSTRG.7714.1	ko04011	STE3; pheromone receptor
	MSTRG.5365.1	ko04011	STE3; pheromone receptor
	MSTRG.10559.1	ko04011	SKO1, ATF1, PCR1; ATF/CREB family transcription factor
	MSTRG.1905.1	ko04011	TEAD; transcriptional enhancer factor
	MSTRG.3829.1	ko04011	GRE2; NADPH-dependent methylglyoxal reductase
	MSTRG.5584.1	ko04011	ROM1_2; RHO1 GDP-GTP exchange protein 1/2
	g5646.t1	ko04010	PKA; protein kinase A
	MSTRG.7679.1	ko04016	ANP1; mitogen-activated protein kinase kinase kinase ANP1
UV-128	MSTRG.10559.1	ko04011	SKO1, ATF1, PCR1; ATF/CREB family transcription factor
	MSTRG.10940.1	ko04011	STE3; pheromone receptor
	MSTRG.5365.1	ko04011	STE3; pheromone receptor
	MSTRG.11179.1	ko04011	STE3; pheromone receptor
	MSTRG.10971.1	ko04011	CSNK1, CKI; casein kinase 1
	MSTRG.7679.1	ko04016	ANP1; mitogen-activated protein kinase kinase kinase ANP1

MAPK signaling pathway—yeast (ko04011), classical MAPK signaling pathway (ko04010), MAPK signaling pathway—plant (ko04016).

**Table 2:** Function annotation of the upregulated DEGs in the MAPK signaling pathway.

Strains	Transcript ID	Pathway ID	Function Description
UV-70	MSTRG.472.1	ko04011	TEAD; transcriptional enhancer factor
	MSTRG.2205.1	ko04011	BCK1; mitogen-activated protein kinase kinase kinase
	MSTRG.4611.1	ko04010	PPP5C; serine/threonine-protein phosphatase 5
UV-104	MSTRG.858.1	ko04011	CSNK1, CKI; casein kinase 1
	MSTRG.5445.1	ko04011	CSNK1, CKI; casein kinase 1
	MSTRG.472.1	ko04011	TEAD; transcriptional enhancer factor
	MSTRG.2758.1	ko04011	NEDD4, RSP5; E3 ubiquitin-protein ligase NEDD4
	MSTRG.2205.1	ko04011	BCK1; mitogen-activated protein kinase kinase kinase
	MSTRG.4611.1	ko04010	PPP5C; serine/threonine-protein phosphatase 5
UV-128	MSTRG.472.1	ko04011	TEAD; transcriptional enhancer factor
	MSTRG.2205.1	ko04011	BCK1; mitogen-activated protein kinase kinase kinase
	MSTRG.4611.1	ko04010	PPP5C; serine/threonine-protein phosphatase 5

MAPK signaling pathway—yeast (ko04011), classical MAPK signaling pathway (ko04010).

**Table 3:** Putative transcription factor and cis-acting element prediction of the hydrophobins.

Transcript IDs	Start Site	Putative TF	Putative Binding Motif
*MSTRG.6832.1*	291 bp	Tec1	CATTCTC
	379 bp	STE12	GAAAACA
*MSTRG.7580.1*	744 bp	Tec1	CATTCTC
	225 bp	STE12	GGAAAGA
*MSTRG.8862.1*	269 bp	Tec1	AATTCTT
	582 bp	STE12	GGAAACA

TEAD family proteins bind strongly to the consensus sequence ANATDCHN in higher eukaryotes or CATTCTT in yeast [50,54], and Ste12 proteins bind to the consensus sequence TGAAACA [55]. The numbers in item Start site mean the distance between the putative binding site and the transcription start site.

**Table 4:** Targeted hydrophobin genes by Tec1 and Ste12.

Transcription Factors	Peak Numbers	Peak Location and Targeted Genes	Gene Function
Tec1	Peak_5307	g2660:Promoter_0kb_1kb	fungal hydrophobin
	Peak_5308	g2661:Promoter_0kb_1kb	fungal hydrophobin
	Peak_1278	g9642:Promoter_0kb_1kb	hydrophobin
	Peak_7599	g4117:Promoter_1kb_2kb	hydrophobin
	Peak_9537	g718:Promoter_0kb_1kb	hydrophobin
	Peak_11029	g6319:Promoter_0kb_1kb	hydrophobin
	Peak_11031	g6321:Promoter_0kb_1kb	hydrophobin
	Peak_2402	g10327:Promoter_0kb_1kb	putative hydrophobin
	Peak_5832	g372:Promoter_0kb_1kb	putative hydrophobin
	Peak_3079	g1973:Promoter_0kb_1kb	putative hydrophobin 2
Ste12	Peak_1356	g9642:Promoter_0kb_1kb	hydrophobin
	Peak_2309	g10197:Promoter_0kb_1kb	hydrophobin
	Peak_5311	g348:Promoter_1kb_2kb	hydrophobin
	Peak_7935	g4117:Promoter_1kb_2kb	hydrophobin
	Peak_11452	g6319:Promoter_0kb_1kb	hydrophobin
	Peak_11454	g6321:Promoter_0kb_1kb	hydrophobin
	Peak_2551	g10327:Promoter_0kb_1kb	putative hydrophobin
	Peak_3238	g1973:Promoter_0kb_1kb	putative hydrophobin 2
	Peak_5575	g2660:Promoter_0kb_1kb	fungal hydrophobin
	Peak_5576	g2661:Promoter_0kb_1kb	fungal hydrophobin

Peak means signal peak where the transcription factor may recognize and bind. In this table, hydrophobin genes whose promoter regions were targeted by signal peaks were chosen.

## Data Availability

RNA-seq rawreads and DAP-seq rawreads are deposited in NCBI SRA with accessions SRR37715099 and SRR37543589, respectively. More data are available on request.

## Supplementary Materials

The following supporting information can be downloaded at: https://www.mdpi.com/article/10.3390/jof12040268/s1, including Supplementary Material S1, Supplementary Material S2, Supplementary Material S3, Supplementary Material S4, information of RNA sequencing, and optical microscopy analysis.
