# Supplementary material for: MAPK Signaling Pathway May Directly Regulate the Expression of Hydrophobin Genes in Flammulina filiformis
Source: J Fungi (Basel). 2026 Apr 8;12(4):268. doi: 10.3390/jof12040268 (PMC13117471; doi:10.3390/jof12040268)
Supplement: Supplementary file 1 [file jof-12-00268-s001.zip › information of RNA sequencing.pdf]

RNA sequencing quality of all samples

Sample	Raw Reads	Clean Reads	Raw Base(G)	Clean Base(G)	Effective Rate(%)	Q20(%)	Q30(%)	GC Content(%)
UV-70-1	18 934 579	18 555 603	5.68	5.57	98	97.16	92.44	53.3
UV-70-2	20 435 542	20 074 984	6.13	6.02	98.24	97.03	92.22	53.26
UV-70-3	19 922 572	19 483 379	5.98	5.85	97.8	96.95	92.06	53.28
UV-104-1	15 577 350	15 237 091	4.67	4.57	97.82	97.01	92.17	53.21
UV-104-2	19 749 784	19 367 297	5.92	5.81	98.06	96.66	91.48	53.11
UV-104-3	21 167 937	20 762 486	6.35	6.23	98.08	97.15	92.4	53.23
UV-128-1	20 319 266	19 896 015	6.1	5.97	97.92	96.91	91.97	53.12
UV-128-2	16 709 410	16 373 558	5.01	4.91	97.99	97.03	92.25	53.2
UV-128-3	17 634 388	17 120 435	5.29	5.14	97.09	97.25	92.59	53.16
WT583-1	20 151 999	19 574 086	6.05	5.87	97.13	97.12	92.4	53.09
WT583-2	15 659 348	15 139 407	4.7	4.54	96.68	97.07	92.31	53.09
WT583-3	17 137 032	16 616 999	5.14	4.99	96.97	97.12	92.38	52.98

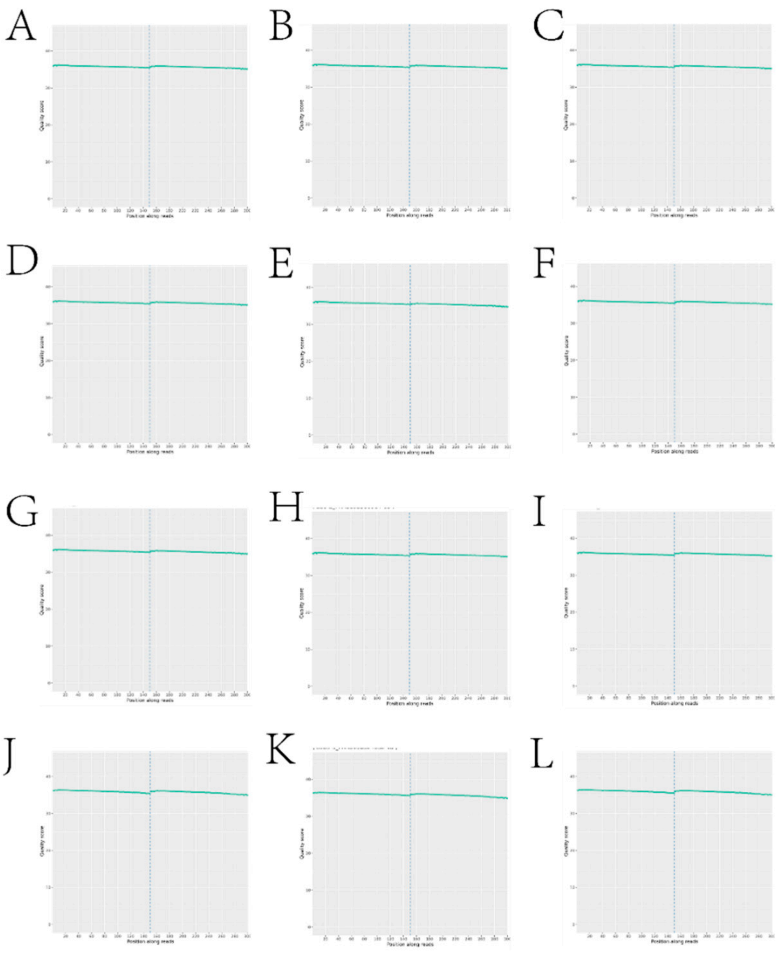

Figure Quality distribution of single base in paired-end reads

First row: WT583; Second row: UV-70; Third row: UV-104; Fourth row:UV-128

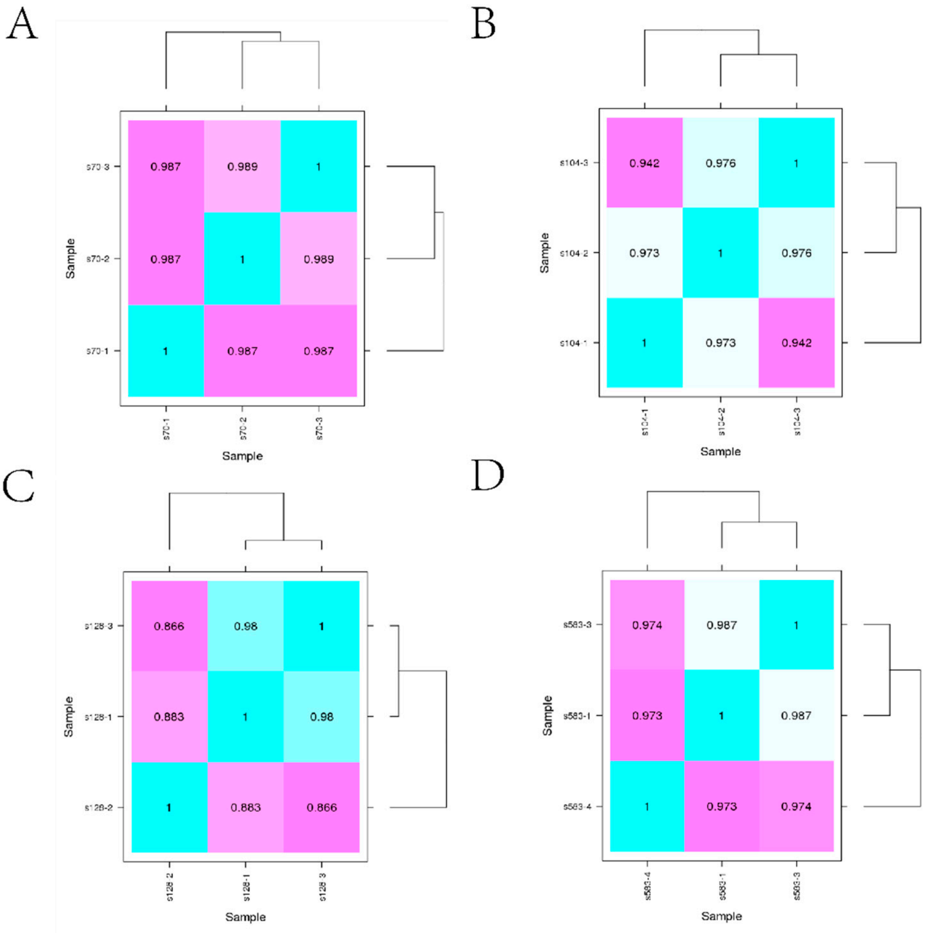

Figure Coorrelation coefficient of sample FPKM

A, B, C, and D represent WT583, UV-70, UV-104, and UV-128, respectively.
