# Supplementary material for: MAPK Signaling Pathway May Directly Regulate the Expression of Hydrophobin Genes in Flammulina filiformis
Source: J Fungi (Basel). 2026 Apr 8;12(4):268. doi: 10.3390/jof12040268 (PMC13117471; doi:10.3390/jof12040268)
Supplement: Supplementary file 1 [file jof-12-00268-s001.zip › supplementary material S1.pdf]

Primers of DEGs in MAPK signaling pathway

Primers	Sequences 5′→3′
6832-F	GTATCATTGCCCTTGGGTGTAG
6832-R	GTATGCAATTGCGTCCAACATC
7562-F	TTCGTCCTGTCACCCATAAAG
7562-R	TATACTTCCCAGGACCTCTACC
7580-F	GTTTGCTGCGAGAACAACAG
7580-R	GAATGACATGGGAACAACGAAC
7655-F	TTAACGTCCAAGACGTGACC
7655-R	GGGATACAGCCAATAGAGATGAG
8862-F	GGAATCGTCCTCCAGGATTTG
8862-R	GATCGGGATGCAACCAATAGA
5365-F	CGTCTATCTTATTCCCGCTTCC
5365-R	GGTGCTTAGAGCTTGACGATAG
7679-F	ACGGGATAACGCCACATTAC
7679-R	TCCTGCATGATCCACATATGAC
10559-F	GCAAGTTCGCAGATGATGAC
10559-R	CTCCTCTGTCTCTGGTTTCTTC
10940-F	GCGCTTAACTACTTGCCATAC
10940-R	GACTAGCCTTTCTACTGCTTCA
10971-F	GGATCAGGCCTCCATCATCTA
10971-R	GTCGTCTCTACGACTAGCATCT
11179-F	CACCCTCGACGTCTTCAATATC
11179-R	TACAATGTCGTGAGGAGGTTTC
472-F	GTTGACGGCAAGACTCTACTAC
472-R	AAGAGAATGAGGTCGTGTTAGC
2205-F	CCACCCTACAGACAACGATAAC
2205-R	TGCCATGGAGATTCCACATC
4611-F	CCAGGCCAAGAAGGACTTATG
4611-R	CCGCTAACGCCATTAAGTGTA
GPD-F	GTTTCCGTTGTTGACCTTGTGTGCC
GPD-R	CGAAGTTGCCGTTGAGCTGGATAC
