# Supplementary material for: MAPK Signaling Pathway May Directly Regulate the Expression of Hydrophobin Genes in Flammulina filiformis
Source: J Fungi (Basel). 2026 Apr 8;12(4):268. doi: 10.3390/jof12040268 (PMC13117471; doi:10.3390/jof12040268)
Supplement: Supplementary file 1 [file jof-12-00268-s001.zip › supplementary material S4.pdf]

Promoter region of Tec1, 1000 bp upstream the translation start site

GTTCTTGAAGAGGTGCGTATGAACATGCATATAAATACCCGCGGGATTTTCCGGGTCTT
GTTCGTTTTTGAGAACAATGGACACCACACCTTCGGTATGTCTTCGCGGTCACGGATCC
GATCTAGTCCATTTGGCGATCTTGTTTTCGTGAGCCTGAGTGTTTTCTTCTCTTCGTTGC
CACAAAACAGGGAAGAATACTGTGCCGGAGGACTCGCTTCCCGGGACTTGGGTCGTT
TTGACCACTGCAAGGAGTCGAAGTTACGAGGCATGAGCCTTGTACCGCGTCACAAAG
ATGCTGCACAGTACAGCATCCGGCGCAAAGGAGCTCGAGGCCTCGAGGGCTCGGCCA
TGCTATGATGCTTCGAGCGTTGATGAATTCAAACAACGGAGACTCTAAGACGGACGTT
CAGGATCCATCACCTCCCTGGGTGAGTCATCCGATTCATTGACATCGGATAGATCTGTT
GACGACTGTTATTACAGGC***CAATTCTC***AACCTCCGGAGATCTGCAGGGTTCGCAATACC
AAGACACCAATTCCACTAAAACTGAACTTTTGTGAGTGTTATTCCTTTGCCATCGAATG
CGGATAGGCGGGGAGCGCTCTTCGTGCTTGAAGCACCCGAAAACTTTCGCTCAAGTTG
TGCTAGCGAGCGCGCGGCTGACAGTCTCTCTGATTAGGCTTCGAT***CCATTCTT***GCGATT
GTCAGTCTACCACGTTTTCCTAAATCGAACGCTGCGCCCCAAGACGGGTTAGTACCTAT
CTTGCCGAGTCGAACTGCTTCGGCGTTTGAATTCAACCTTTTCATTCAGAACCTTACCC
AGTTAAGATCGAACTTTACGAGATTCGGGTGGCAGATGTCTGGGAGCCGACTTAGGGC
TGCCTTAGACAATTCTGATGCAGTGATGGTATTGTGCGAAGATCCTTTAAAGGTCTGCC
TAAACGCTTCATAAAGTGTGTGCCCACACTCTTTTCCCCTCACTCCTTCCGTCTACCTTT
ATCC
